# Supplementary material for: Heparin-based hydrogel scaffolding alters the transcriptomic profile and increases the chemoresistance of MDA-MB-231 triple-negative breast cancer cells
Source: Biomater Sci. 2020 Feb 13;8(10):2786–96. doi: 10.1039/c9bm01481k (PMC7497406; doi:10.1039/c9bm01481k)
Supplement: Supplementary file 2 [file BM-008-C9BM01481K-s002.zip › Supplementary File 4/HP-B hydrogel + EGF (s)vControl/Pathways/my_analysis.Gsea.1545201170987/HALLMARK_G2M_CHECKPOINT.html]

Details for gene set HALLMARK\_G2M\_CHECKPOINT[GSEA]

|  || Dataset | expr.class.cls#HYDEGF\_versus\_CONTROL.class.cls#HYDEGF\_versus\_CONTROL\_repos |
| Phenotype | class.cls#HYDEGF\_versus\_CONTROL\_repos |
| Upregulated in class | HYDEGF |
| GeneSet | HALLMARK\_G2M\_CHECKPOINT |
| Enrichment Score (ES) | 0.34513587 |
| Normalized Enrichment Score (NES) | 1.5773185 |
| Nominal p-value | 0.0 |
| FDR q-value | 0.024639413 |
| FWER p-Value | 0.151 |
Table: GSEA Results Summary

  

Fig 1: Enrichment plot: HALLMARK\_G2M\_CHECKPOINT      
 Profile of the Running ES Score & Positions of GeneSet Members on the Rank Ordered List

  

| PROBE | DESCRIPTION (from dataset) | GENE SYMBOL | GENE\_TITLE | RANK IN GENE LIST | RANK METRIC SCORE | RUNNING ES | CORE ENRICHMENT || 1 | DDX39A | na |  |  | 115 | 2.860 | 0.0126 | Yes |
| 2 | EGF | na |  |  | 131 | 2.796 | 0.0300 | Yes |
| 3 | AURKB | na |  |  | 223 | 2.534 | 0.0417 | Yes |
| 4 | SLC7A5 | na |  |  | 239 | 2.501 | 0.0573 | Yes |
| 5 | TACC3 | na |  |  | 271 | 2.421 | 0.0714 | Yes |
| 6 | RBM14 | na |  |  | 342 | 2.322 | 0.0829 | Yes |
| 7 | E2F1 | na |  |  | 358 | 2.297 | 0.0970 | Yes |
| 8 | POLQ | na |  |  | 374 | 2.267 | 0.1110 | Yes |
| 9 | ESPL1 | na |  |  | 387 | 2.248 | 0.1250 | Yes |
| 10 | CCNF | na |  |  | 443 | 2.181 | 0.1364 | Yes |
| 11 | RAD54L | na |  |  | 617 | 1.992 | 0.1402 | Yes |
| 12 | E2F2 | na |  |  | 687 | 1.916 | 0.1491 | Yes |
| 13 | MCM2 | na |  |  | 778 | 1.847 | 0.1564 | Yes |
| 14 | CDKN2C | na |  |  | 781 | 1.845 | 0.1683 | Yes |
| 15 | TROAP | na |  |  | 855 | 1.781 | 0.1761 | Yes |
| 16 | E2F4 | na |  |  | 1075 | 1.638 | 0.1752 | Yes |
| 17 | CHAF1A | na |  |  | 1100 | 1.622 | 0.1845 | Yes |
| 18 | MYBL2 | na |  |  | 1167 | 1.589 | 0.1914 | Yes |
| 19 | EZH2 | na |  |  | 1241 | 1.545 | 0.1977 | Yes |
| 20 | SRSF10 | na |  |  | 1250 | 1.537 | 0.2072 | Yes |
| 21 | POLA2 | na |  |  | 1347 | 1.495 | 0.2119 | Yes |
| 22 | POLE | na |  |  | 1398 | 1.468 | 0.2189 | Yes |
| 23 | DTYMK | na |  |  | 1429 | 1.457 | 0.2268 | Yes |
| 24 | INCENP | na |  |  | 1487 | 1.437 | 0.2332 | Yes |
| 25 | RASAL2 | na |  |  | 1606 | 1.389 | 0.2360 | Yes |
| 26 | ODF2 | na |  |  | 1613 | 1.387 | 0.2447 | Yes |
| 27 | FANCC | na |  |  | 1650 | 1.374 | 0.2518 | Yes |
| 28 | KIF22 | na |  |  | 1674 | 1.366 | 0.2595 | Yes |
| 29 | GINS2 | na |  |  | 1691 | 1.361 | 0.2675 | Yes |
| 30 | CDC25A | na |  |  | 1707 | 1.357 | 0.2756 | Yes |
| 31 | PAPD7 | na |  |  | 1842 | 1.313 | 0.2771 | Yes |
| 32 | ILF3 | na |  |  | 2042 | 1.253 | 0.2748 | Yes |
| 33 | TNPO2 | na |  |  | 2110 | 1.235 | 0.2793 | Yes |
| 34 | CDC45 | na |  |  | 2137 | 1.228 | 0.2859 | Yes |
| 35 | TRAIP | na |  |  | 2275 | 1.188 | 0.2865 | Yes |
| 36 | KIF15 | na |  |  | 2417 | 1.147 | 0.2865 | Yes |
| 37 | PLK4 | na |  |  | 2424 | 1.146 | 0.2937 | Yes |
| 38 | UPF1 | na |  |  | 2520 | 1.122 | 0.2960 | Yes |
| 39 | SMC1A | na |  |  | 2558 | 1.113 | 0.3013 | Yes |
| 40 | CDC6 | na |  |  | 2609 | 1.102 | 0.3058 | Yes |
| 41 | CCND1 | na |  |  | 2684 | 1.084 | 0.3090 | Yes |
| 42 | MCM5 | na |  |  | 2694 | 1.082 | 0.3156 | Yes |
| 43 | TFDP1 | na |  |  | 2728 | 1.072 | 0.3208 | Yes |
| 44 | ABL1 | na |  |  | 2791 | 1.054 | 0.3244 | Yes |
| 45 | MCM3 | na |  |  | 3325 | 0.938 | 0.3025 | Yes |
| 46 | PRPF4B | na |  |  | 3359 | 0.931 | 0.3069 | Yes |
| 47 | SUV39H1 | na |  |  | 3419 | 0.918 | 0.3097 | Yes |
| 48 | H2AFX | na |  |  | 3433 | 0.916 | 0.3150 | Yes |
| 49 | ATF5 | na |  |  | 3511 | 0.903 | 0.3169 | Yes |
| 50 | SFPQ | na |  |  | 3547 | 0.894 | 0.3208 | Yes |
| 51 | STAG1 | na |  |  | 3553 | 0.892 | 0.3264 | Yes |
| 52 | CENPF | na |  |  | 3556 | 0.892 | 0.3321 | Yes |
| 53 | TPX2 | na |  |  | 3754 | 0.855 | 0.3273 | Yes |
| 54 | SLC7A1 | na |  |  | 3784 | 0.850 | 0.3313 | Yes |
| 55 | UBE2S | na |  |  | 3814 | 0.843 | 0.3353 | Yes |
| 56 | YTHDC1 | na |  |  | 3943 | 0.822 | 0.3339 | Yes |
| 57 | ODC1 | na |  |  | 3955 | 0.820 | 0.3387 | Yes |
| 58 | SLC12A2 | na |  |  | 3959 | 0.820 | 0.3439 | Yes |
| 59 | TRA2B | na |  |  | 4229 | 0.777 | 0.3348 | Yes |
| 60 | NUMA1 | na |  |  | 4302 | 0.766 | 0.3360 | Yes |
| 61 | MYC | na |  |  | 4314 | 0.764 | 0.3404 | Yes |
| 62 | BUB1 | na |  |  | 4319 | 0.763 | 0.3451 | Yes |
| 63 | SMC4 | na |  |  | 4414 | 0.747 | 0.3451 | No |
| 64 | CDC20 | na |  |  | 4567 | 0.724 | 0.3418 | No |
| 65 | RBL1 | na |  |  | 4667 | 0.710 | 0.3412 | No |
| 66 | NUP50 | na |  |  | 4832 | 0.682 | 0.3370 | No |
| 67 | CASP8AP2 | na |  |  | 4925 | 0.665 | 0.3365 | No |
| 68 | MAPK14 | na |  |  | 5197 | 0.620 | 0.3263 | No |
| 69 | NUP98 | na |  |  | 5263 | 0.611 | 0.3269 | No |
| 70 | CDC25B | na |  |  | 5390 | 0.592 | 0.3241 | No |
| 71 | E2F3 | na |  |  | 5528 | 0.572 | 0.3206 | No |
| 72 | CUL1 | na |  |  | 5551 | 0.570 | 0.3232 | No |
| 73 | PRC1 | na |  |  | 5609 | 0.561 | 0.3238 | No |
| 74 | BARD1 | na |  |  | 5616 | 0.559 | 0.3272 | No |
| 75 | KIF23 | na |  |  | 5633 | 0.557 | 0.3299 | No |
| 76 | NOLC1 | na |  |  | 5664 | 0.551 | 0.3320 | No |
| 77 | PRIM2 | na |  |  | 5670 | 0.551 | 0.3353 | No |
| 78 | KIF2C | na |  |  | 5742 | 0.538 | 0.3351 | No |
| 79 | CHMP1A | na |  |  | 5891 | 0.519 | 0.3306 | No |
| 80 | LIG3 | na |  |  | 5968 | 0.508 | 0.3300 | No |
| 81 | DMD | na |  |  | 5983 | 0.504 | 0.3325 | No |
| 82 | SMAD3 | na |  |  | 6056 | 0.496 | 0.3320 | No |
| 83 | EXO1 | na |  |  | 6063 | 0.494 | 0.3349 | No |
| 84 | DBF4 | na |  |  | 6300 | 0.463 | 0.3255 | No |
| 85 | UCK2 | na |  |  | 6380 | 0.452 | 0.3243 | No |
| 86 | BRCA2 | na |  |  | 6406 | 0.449 | 0.3259 | No |
| 87 | WRN | na |  |  | 6663 | 0.415 | 0.3151 | No |
| 88 | CDC7 | na |  |  | 6713 | 0.409 | 0.3152 | No |
| 89 | NOTCH2 | na |  |  | 7202 | 0.343 | 0.2918 | No |
| 90 | TLE3 | na |  |  | 7257 | 0.336 | 0.2911 | No |
| 91 | CHEK1 | na |  |  | 7331 | 0.325 | 0.2894 | No |
| 92 | HMGN2 | na |  |  | 7401 | 0.317 | 0.2878 | No |
| 93 | PLK1 | na |  |  | 7470 | 0.309 | 0.2862 | No |
| 94 | KIF4A | na |  |  | 7539 | 0.300 | 0.2846 | No |
| 95 | CENPE | na |  |  | 7608 | 0.290 | 0.2829 | No |
| 96 | SRSF1 | na |  |  | 7695 | 0.280 | 0.2802 | No |
| 97 | PML | na |  |  | 7928 | 0.252 | 0.2697 | No |
| 98 | STIL | na |  |  | 8443 | 0.196 | 0.2439 | No |
| 99 | CUL4A | na |  |  | 8492 | 0.188 | 0.2426 | No |
| 100 | RACGAP1 | na |  |  | 8576 | 0.177 | 0.2394 | No |
| 101 | NUSAP1 | na |  |  | 8930 | 0.130 | 0.2217 | No |
| 102 | PAFAH1B1 | na |  |  | 8956 | 0.125 | 0.2212 | No |
| 103 | NDC80 | na |  |  | 9060 | 0.121 | 0.2166 | No |
| 104 | BIRC5 | na |  |  | 9147 | 0.110 | 0.2127 | No |
| 105 | LMNB1 | na |  |  | 9148 | 0.110 | 0.2135 | No |
| 106 | AMD1 | na |  |  | 9158 | 0.108 | 0.2137 | No |
| 107 | CBX1 | na |  |  | 9287 | 0.090 | 0.2075 | No |
| 108 | HIRA | na |  |  | 9316 | 0.087 | 0.2066 | No |
| 109 | NCL | na |  |  | 9331 | 0.084 | 0.2064 | No |
| 110 | KPNB1 | na |  |  | 9341 | 0.083 | 0.2065 | No |
| 111 | DKC1 | na |  |  | 9375 | 0.079 | 0.2053 | No |
| 112 | KIF20B | na |  |  | 9759 | 0.042 | 0.1854 | No |
| 113 | PDS5B | na |  |  | 9810 | 0.034 | 0.1830 | No |
| 114 | ARID4A | na |  |  | 9817 | 0.033 | 0.1829 | No |
| 115 | G3BP1 | na |  |  | 9872 | 0.026 | 0.1802 | No |
| 116 | NASP | na |  |  | 10061 | 0.011 | 0.1704 | No |
| 117 | SMC2 | na |  |  | 10167 | -0.004 | 0.1649 | No |
| 118 | ORC6 | na |  |  | 10250 | -0.016 | 0.1607 | No |
| 119 | MCM6 | na |  |  | 10326 | -0.026 | 0.1569 | No |
| 120 | MNAT1 | na |  |  | 10591 | -0.049 | 0.1434 | No |
| 121 | BUB3 | na |  |  | 10780 | -0.077 | 0.1340 | No |
| 122 | CDKN3 | na |  |  | 10895 | -0.093 | 0.1286 | No |
| 123 | CUL5 | na |  |  | 10984 | -0.104 | 0.1246 | No |
| 124 | SNRPD1 | na |  |  | 11061 | -0.116 | 0.1214 | No |
| 125 | KIF5B | na |  |  | 11200 | -0.123 | 0.1149 | No |
| 126 | CCNB2 | na |  |  | 11220 | -0.126 | 0.1147 | No |
| 127 | KIF11 | na |  |  | 11269 | -0.132 | 0.1131 | No |
| 128 | AURKA | na |  |  | 11282 | -0.134 | 0.1133 | No |
| 129 | CCNA2 | na |  |  | 11402 | -0.146 | 0.1080 | No |
| 130 | MEIS1 | na |  |  | 11548 | -0.167 | 0.1015 | No |
| 131 | MTF2 | na |  |  | 11691 | -0.184 | 0.0952 | No |
| 132 | ATRX | na |  |  | 11758 | -0.197 | 0.0930 | No |
| 133 | PRMT5 | na |  |  | 12050 | -0.224 | 0.0792 | No |
| 134 | EWSR1 | na |  |  | 12144 | -0.236 | 0.0758 | No |
| 135 | CUL3 | na |  |  | 12229 | -0.250 | 0.0730 | No |
| 136 | CDK4 | na |  |  | 12236 | -0.251 | 0.0744 | No |
| 137 | LBR | na |  |  | 12297 | -0.261 | 0.0729 | No |
| 138 | H2AFZ | na |  |  | 12572 | -0.292 | 0.0604 | No |
| 139 | TOP2A | na |  |  | 12732 | -0.319 | 0.0541 | No |
| 140 | FOXN3 | na |  |  | 12793 | -0.327 | 0.0531 | No |
| 141 | XPO1 | na |  |  | 13014 | -0.343 | 0.0437 | No |
| 142 | TTK | na |  |  | 13159 | -0.362 | 0.0385 | No |
| 143 | GSPT1 | na |  |  | 13252 | -0.378 | 0.0361 | No |
| 144 | HMMR | na |  |  | 13307 | -0.387 | 0.0358 | No |
| 145 | ORC5 | na |  |  | 13393 | -0.402 | 0.0340 | No |
| 146 | NEK2 | na |  |  | 13399 | -0.403 | 0.0363 | No |
| 147 | CTCF | na |  |  | 13593 | -0.423 | 0.0289 | No |
| 148 | FBXO5 | na |  |  | 13612 | -0.427 | 0.0308 | No |
| 149 | SLC38A1 | na |  |  | 13647 | -0.433 | 0.0318 | No |
| 150 | DR1 | na |  |  | 13808 | -0.460 | 0.0264 | No |
| 151 | TMPO | na |  |  | 13866 | -0.469 | 0.0264 | No |
| 152 | TGFB1 | na |  |  | 14260 | -0.515 | 0.0091 | No |
| 153 | CENPA | na |  |  | 14298 | -0.521 | 0.0106 | No |
| 154 | CCNT1 | na |  |  | 14554 | -0.565 | 0.0008 | No |
| 155 | RPS6KA5 | na |  |  | 14558 | -0.566 | 0.0044 | No |
| 156 | PURA | na |  |  | 14580 | -0.572 | 0.0070 | No |
| 157 | HNRNPU | na |  |  | 14621 | -0.576 | 0.0086 | No |
| 158 | CKS1B | na |  |  | 14664 | -0.584 | 0.0102 | No |
| 159 | SRSF2 | na |  |  | 14778 | -0.601 | 0.0082 | No |
| 160 | MEIS2 | na |  |  | 14981 | -0.630 | 0.0017 | No |
| 161 | MAD2L1 | na |  |  | 15013 | -0.635 | 0.0042 | No |
| 162 | SS18 | na |  |  | 15190 | -0.667 | -0.0007 | No |
| 163 | RAD21 | na |  |  | 15296 | -0.686 | -0.0018 | No |
| 164 | MARCKS | na |  |  | 15423 | -0.704 | -0.0038 | No |
| 165 | CDKN1B | na |  |  | 15677 | -0.758 | -0.0122 | No |
| 166 | RAD23B | na |  |  | 15689 | -0.759 | -0.0078 | No |
| 167 | UBE2C | na |  |  | 15891 | -0.800 | -0.0132 | No |
| 168 | CDK1 | na |  |  | 15956 | -0.811 | -0.0113 | No |
| 169 | SMARCC1 | na |  |  | 15991 | -0.816 | -0.0077 | No |
| 170 | SYNCRIP | na |  |  | 16017 | -0.824 | -0.0037 | No |
| 171 | PBK | na |  |  | 16119 | -0.850 | -0.0034 | No |
| 172 | RPA2 | na |  |  | 16249 | -0.886 | -0.0045 | No |
| 173 | H2AFV | na |  |  | 16307 | -0.900 | -0.0016 | No |
| 174 | KPNA2 | na |  |  | 16498 | -0.947 | -0.0054 | No |
| 175 | MT2A | na |  |  | 16590 | -0.967 | -0.0039 | No |
| 176 | SQLE | na |  |  | 16592 | -0.967 | 0.0023 | No |
| 177 | KATNA1 | na |  |  | 16922 | -1.062 | -0.0080 | No |
| 178 | PTTG1 | na |  |  | 16987 | -1.085 | -0.0043 | No |
| 179 | CDC27 | na |  |  | 17136 | -1.124 | -0.0048 | No |
| 180 | HUS1 | na |  |  | 17253 | -1.162 | -0.0033 | No |
| 181 | HIF1A | na |  |  | 17317 | -1.190 | 0.0011 | No |
| 182 | BCL3 | na |  |  | 17353 | -1.200 | 0.0071 | No |
| 183 | EFNA5 | na |  |  | 17521 | -1.267 | 0.0066 | No |
| 184 | STMN1 | na |  |  | 17644 | -1.311 | 0.0087 | No |
| 185 | SAP30 | na |  |  | 17712 | -1.340 | 0.0139 | No |
| 186 | HIST1H2BK | na |  |  | 17754 | -1.361 | 0.0206 | No |
| 187 | TOP1 | na |  |  | 18213 | -1.609 | 0.0070 | No |
| 188 | HNRNPD | na |  |  | 18418 | -1.769 | 0.0078 | No |
| 189 | CKS2 | na |  |  | 18969 | -2.501 | -0.0048 | No |
| 190 | HMGB3 | na |  |  | 19000 | -2.585 | 0.0104 | No |
Table: GSEA details [plain text format]

  

Fig 2: HALLMARK\_G2M\_CHECKPOINT      
 Blue-Pink O' Gram in the Space of the Analyzed GeneSet

  

Fig 3: HALLMARK\_G2M\_CHECKPOINT: Random ES distribution      
 Gene set null distribution of ES for **HALLMARK\_G2M\_CHECKPOINT**
